# Monitoring and early warning of ovarian cancer using high-dimensional non-parametric EWMA control chart based on sliding window

**DOI:** 10.1038/s41598-025-86576-w

**Published:** 2025-03-17

**Authors:** Bin Wu, Wen Zhong, Yixing Ren, Zhongli Zhou, Liu Liu

**Affiliations:** 1 College of Management Science, Chengdu University of Technology, Chengdu, 610059 China; 2 School of Big Data and Statistics, Sichuan Tourism College, Chengdu, 610100 China; 3 Department of Gastroenterology, Affiliated Hospital of North Sichuan Medical College, Nanchong, 637000 China; 4 College of Mathematics and Physics and Geomathematics Key Laboratory of Sichuan Province, Chengdu University of Technology, Chengdu, 610059 China

**Keywords:** Statistical process control, Benign ovarian tumors, Ovarian cancer, Empirical likelihood ratio test, EWMA, Cysts, Tumour biomarkers, Biomarkers, Diseases, Health care, Oncology, Mathematics and computing

## Abstract

Ovarian tumors are a common ovarian dysfunction that affects women’s daily lives. Although ovarian tumors are generally sensitive to chemotherapy and initially respond well to platinum/taxane-based treatments, the postoperative recurrence rate remains high in advanced cases. Many researchers are dedicated to developing new methods for monitoring and predicting malignant tumors. Traditional approaches use dimensionality reduction techniques, like principal component analysis and deep learning, to select relevant features, followed by univariate or multivariate control charts for monitoring. However, these methods may overlook interactions between features and dimensionality reduction can result in loss of information, potentially affecting the accuracy of the model and leading to delayed alerts and reduced predictive performance. Therefore, this paper develops a new sliding window EWMA control chart based on high-dimensional empirical likelihood ratio tests. This control chart not only monitors data with unknown underlying distributions but is also applicable to high-dimensional data, allowing for monitoring without dimensionality reduction, thus simplifying the process and avoiding information loss. Monte Carlo results show that this method detects changes in indicators and issues alerts more rapidly than the dimensionality-reduced multivariate EWMA control charts. In addition, we further validated the effectiveness of this method through analysis of a tumor resection data example.

**Supplementary Information:**

The online version contains supplementary material available at 10.1038/s41598-025-86576-w.

## Introduction

Ovarian tumors are a prevalent form of ovarian dysfunction in women, typically appearing as cyst-like masses in one or both ovaries. While the majority of ovarian cysts are benign, there is a risk that some may undergo malignant transformation, leading to the development of cancer. During a woman’s lifetime, the risk of developing ovarian cancer is 1 in 75, and the risk of dying from this deadly disease is 1 in 100^1^. The incidence of the disease varies by age and ethnicity, with a higher incidence (approximately 70%) in less developed countries^2^. In 2018, the global death rate from ovarian cancer was 4.4%. In 2020, there were approximately 21,750 new cases of ovarian cancer in the United States, resulting in about 13,940 deaths from the disease^3,4^. Due to the lack of early symptoms of ovarian cancer, and even when symptoms are present, they are often nonspecific. As a result, the effectiveness of screening is limited, making early diagnosis challenging. By the time patients seek medical attention, 60–70% of cases are already in advanced stages, with the mortality rate for advanced ovarian cancer reaching as high as 60–80%^5^. Although ovarian cancer demonstrates initial sensitivity to chemotherapy, particularly with platinum/taxane regimens, the rate of recurrence within five years for patients with advanced disease remains high, ranging from 60 to 80%. Recurrence significantly jeopardizes patient survival^6^. Ovarian cancer, while less prevalent than cervical and endometrial cancers and ranking third among gynecological malignancies, has a mortality rate that exceeds the sum of these two, positioning it as the foremost cause of death from gynecological cancers and a significant threat to women’s health^7,8^. Therefore, it is crucial to conduct online surveillance of ovarian cyst data to promptly detect and alert changes in relevant biomarkers, thereby preventing the progression and potential advancement of the tumor to an advanced stage.

For the diagnosis of ovarian cancer in patients, clinical assessments often extend beyond physical examination, which includes pelvic examination and vaginal ultrasound, to the evaluation of certain serum biomarkers, such as CA-125 and HE4. Numerous studies have been undertaken to assess the efficacy of these biomarkers. Anton et al.^9^evaluated the expression of CA-125 and HE4 in 128 patients diagnosed with ovarian masses using imaging analysis, with findings indicating that HE4 demonstrated the highest overall sensitivity. Hou et al. utilized the Abbott Architect i2000SR to measure preoperative serum marker levels across groups categorized by ovarian cancer, ovarian benign tumors, and normal controls. Their results indicated that, with the exception of HE4 (76.67%) and CA-125 (68.33%), ovarian cancer tumor markers generally exhibited high diagnostic specificity but low sensitivity^10^. Vincent Dochez et al.^11^. analyzed the sensitivity of CA-125 and HE4 in a cohort of 221 patients, concluding that the combined HE4 and CA-125 assay is the most effective predictor of ovarian cancer risk in patients with a pelvic mass of unknown origin (PBOT). Collectively, current clinical diagnostic strategies for ovarian cancer (OC) are centered around a limited number of biomarkers, selected based on gene/protein overexpression or supported by epidemiological data^12^. It is anticipated that the application of statistical methodologies can enhance the monitoring and early warning of malignancy in patients with suspected ovarian tumors.

Statistical process control (SPC) is a quantitative and scientific method of quality control that utilizes mathematical statistics and big data analysis techniques to monitor and alert risks at each stage of a process, with the aim of improving and ensuring quality control. In recent years, an increasing number of SPC methods have been applied in medical research. One of the most prevalent areas of research and application is the development of a series of risk-adjusted models. For instance, Rafieid et al.^13^ proposed a risk-adjusted control chart for healthcare systems that incorporates both economic and statistical factors. Asif et al.^14^introduced a risk-adjusted moving average-exponentially weighted moving average (RAMA-EWMA) control chart for estimating survival time following cardiac surgery. Additional recent studies on risk-adjusted models can be found in references^15–17^. In addition, Rashed et al.^18^ conducted long-term monitoring of cancer mortality in the United States using statistical analysis and SPC methods. Li et al.^19^ assessed the effectiveness of surface-guided radiation therapy (SGRT) in clinical applications by constructing exponentially weighted moving average (EWMA) control charts. Riis et al.^20^employed multivariate SPC to screen prostate cancer patients. For more applications of control charts in the medical field, please refer to references^21–23^. However, it is worth noting that while these studies have successfully applied SPC methods to monitor and provide early warnings for various diseases, they either use one-dimensional control charts to monitor individual features separately, which can be time-consuming and resource-intensive, or they employ traditional dimensionality reduction techniques (such as PCA, LASSO, deep learning, etc.) to reduce data dimensions before using multivariate control charts for monitoring. However, these methods may exclude key variables associated with anomalous information, leading to the omission of critical details and affecting the accuracy of the model, resulting in delayed alarms and reduced predictive performance. Additionally, PCA dimensionality reduction typically combines original variables into new variables through linear combinations, which often results in less clear and accurate interpretations compared to the original variables. Given that medical data are often complex, high-dimensional, and lack known underlying distributions, there is a need to develop novel control charts suitable for this type of data. Therefore, traditional one-dimensional or multidimensional parameter control charts^24–29^are no longer applicable. Furthermore, while some researchers have proposed high-dimensional nonparametric control charts^30,31^, their methods are designed for subgroup data flow, requiring grouped data monitoring (each group typically containing more than 5 samples), which is not suitable for small-sample ovarian cyst data. Consequently, this paper proposes using nonparametric high-dimensional empirical likelihood ratio tests^32^ to address high-dimensional and non-parametric data challenges, combined with EWMA control charts for online monitoring. Additionally, we will utilize a sliding window approach for single-point data monitoring to achieve effective online monitoring of ovarian tumor data.

The remainder of this paper is organized as follows: Sect. 2 provides a detailed description of the proposed control chart. Section 3 presents a simulation study to evaluate the performance of the method. Section 4 applies the proposed method to real-world data. Finally, Sect. 5 summarizes the conclusions.

## Statistical methods

### Empirical likelihood ratio test

The empirical likelihood ratio test, recognized as a potent nonparametric statistical inference tool, has undergone significant development in statistical inference and found applications across various domains. Initially introduced by Owen^33,34^, empirical likelihood offers a compelling advantage by enabling likelihood inference in nonparametric or semi-parametric scenarios, resembling traditional likelihood functions that adhere to Wilks’ theorem and Bartlett’s adjustment.

Suppose $\:{x}_{1},\:{x}_{2},\cdots\:,{x}_{n}$ are independent and identically distributed samples drawn from a -dimensional population with mean vector $\:\mu\:$ and covariance matrix $\:\varSigma\:$. The traditional empirical likelihood ratio test is defined as

1 $$\:R\left(\mu\:\right)=\text{m}\text{a}\text{x}\left\{\prod\:_{i=1}^{n}n{p}_{i}\right|{p}_{i}\ge\:0,\sum\:_{i=1}^{n}{p}_{i}=1,\:\sum\:_{i=1}^{n}{p}_{i}{x}_{i}=\mu\:\}$$,

Where $\:{p}_{i}=F\left({x}_{i}\right)-F\left({x}_{i}-\right),\:\:F\left(x\right)={n}^{-1}{\sum\:}_{i=1}^{n}I({x}_{i}\le\:x)$ is the empirical distribution function, $\:I(\bullet\:)$ is the indicative function, which means that when $\:{x}_{i}\le\:x,\:\:I\left({x}_{i}\le\:x\right)=1$, otherwise 0.

Extending the traditional empirical likelihood method to statistical inference for high-dimensional data faces two primary challenges. Firstly, there’s the concern of whether the sample convex hull, denoted by $\:{x}_{i}-\mu\:,\:i=1,\:2,\:\cdots\:,\:n$ encompasses the zero vector. Tsao et al.^35^ noted that when the sample dimension *p* and sample size *n* satisfy $\:p/n>1/2$, the probability of the convex hull covering the zero vector approaches zero, rendering the equation $\:{\sum\:}_{i=1}^{n}{p}_{i}{x}_{i}=\mu\:$ with no solution. Secondly, there’s the issue of singularity in the covariance matrix. In response to these challenges, various scholars have proposed solutions. For instance, Chen et al.^36^ suggested augmenting the *n* sample points with an artificial virtual point to mitigate the absence of solutions in $\:{\sum\:}_{i=1}^{n}{p}_{i}{x}_{i}=\mu\:$. Emerson et al.^37^, building upon Chen’s approach, introduced a balancing point to maintain the original mean. However, the virtual points added by Chen and Emerson rely on the inverse of the sample covariance matrix and are inapplicable when the sample dimension *p* exceeds the sample size *n*. Cui et al.^32^ has improved on the basis of Chen and Emerson, so that the added two artificial virtual points no longer contain the inverse of the sample covariance, and the proposed test method can be extended to high-dimensional statistical inference. The artificial virtual points introduced are defined as follows:

2 $$\:{x}_{n+1}={\mu\:}_{0}-{a}_{n}\left(\stackrel{-}{x}-{\mu\:}_{0}\right),\:{\:x}_{n+2}={\mu\:}_{0}+(2+{a}_{n})\left(\stackrel{-}{x}-{\mu\:}_{0}\right)$$

Where $\:{a}_{n}=\frac{{l}_{n}}{{\{{||\stackrel{-}{x}-{\mu\:}_{0}||}^{2}+{k}_{n}|{\gamma\:}^{T}{(\stackrel{-}{x}-{\mu\:}_{0})}^{2}\left|\right\}}^{1/2}}$, with $\:{a}_{n}$ depending on positive constant $\:{k}_{n}$ and $\:{l}_{n}$. Here $\:\gamma\:$ is a p-dimensional constant vector that satisfying $\:\left|\left|\gamma\:\right|\right|=1$.

The enhanced empirical likelihood ratio test is as follows:

3 $$\:R\left(\mu\:,{k}_{n}\right)=\text{m}\text{a}\text{x}\left\{\prod\:_{i=1}^{n+2}(n+2){p}_{i}\right|{p}_{i}\ge\:0,\sum\:_{i=1}^{n+2}{p}_{i}=1,\:\sum\:_{i=1}^{n+2}{p}_{i}{(x}_{i}-{\mu\:}_{0})=0\}$$

### EWMA control chart

In 1959, Roberts et al.^38^ proposed EWMA control charts, leveraging historical data while prioritizing the significance of current samples and progressively diminishing the influence of past observations. This approach proves effective in monitoring mean value shifts. By integrating historical and current observations and flexibly adjusting parameters, EWMA charts offer a balanced monitoring performance across different magnitudes of process shifts. Assuming that the mass eigenvalue *x* is independent and follows a normal distribution, i.e., $\:x{\sim}N(\mu\:,{\sigma\:}^{2})$, the statistic for the EWMA control chart is defined as:

$$\:{Z}_{i}=\left(1-\lambda\:\right){Z}_{i-1}+\lambda\:{x}_{i},$$

where the constant $\:\lambda\:\in\:\left(\text{0,1}\right]$ serves as a smoothing parameter. Generally, for EWMA-type control charts, selecting a relatively small $\:\lambda\:$enhances the detection of minor shifts^38^, whereas opting for a relatively large $\:\lambda\:$ proves more effective in identifying substantial shifts.

### EWMA control chart with sliding window based on high-dimensional empirical likelihood

The non-parametric high-dimensional empirical likelihood ratio statistic proposed by Cui et al.^32^ is

4 $$\:{Q}_{n}={\left\{2\widehat{tr}\right({{\Omega\:}}^{2}\left)\right\}}^{-1/2}\left\{\frac{2n{l}_{n}^{2}}{{\left(n+2\right)}^{2}}W\left({\mu\:}_{0},{k}_{n}\right)-\widehat{tr}\left({\Omega\:}\right)\right\}$$

Where $\:{\Omega\:}={{\Sigma\:}}^{\frac{1}{2}}({I}_{p}+{k}_{n}\gamma\:{\gamma\:}^{T}){{\Sigma\:}}^{1/2}$, $\:{I}_{p}$ is $\:p\times\:p$ identity matrix. $\:tr(\bullet\:)$ is the trace of a matrix. $\:\widehat{tr}(\bullet\:)$ is the estimate of $\:tr(\bullet\:)$. And $\:W({\mu\:}_{0},{k}_{n})$ represents the expression derived for $\:-2log\left(R\right({\mu\:}_{0},{k}_{n}\left)\right)$.

Let $$\:{\xi\:}_{n}=\frac{n+2}{1+{a}_{n}}$$, then

$$\:W\left({\mu\:}_{0},{k}_{n}\right)=-2(nlog\left[1+\frac{1}{n}\left\{1-{\left(1+\frac{n}{n+2}{\xi\:}_{n}^{2}\right)}^{\frac{1}{2}}\right\}\right]$$

5 $$\:+\text{log}\left\{\frac{1}{2}+\frac{{\xi\:}_{n}}{2}+\frac{1}{2}{\left(1+\frac{n}{n+2}{\xi\:}_{n}^{2}\right)}^{\frac{1}{2}}\right\}\:+\text{log}\left\{\frac{1}{2}-\frac{{\xi\:}_{n}}{2}+\frac{1}{2}{\left(1+\frac{n}{n+2}{\xi\:}_{n}^{2}\right)}^{\frac{1}{2}}\right\}$$

Our approach involves integrating the proposed high-dimensional empirical likelihood ratio test with EWMA control charts to construct monitoring statistics. To facilitate single data stream monitoring, we introduce a sliding window *D*. As the detection point shifts to the subsequent position, a new sample point replaces the one at the furthest position within the window, thus constituting a fresh detection window. The monitoring statistic, hereafter referred to as D-ELEWMA, is formulated as follows:

6 $$\:{T}_{t}^{D}=\left(1-\lambda\:\right){T}_{t-1}^{D}+\lambda\:{Q}_{t}^{D},\:(0<\lambda\:<1)$$

Let $\:{X}_{i}=({x}_{i1},{x}_{i2},\cdots\:,{x}_{ip}){\prime\:}$ denote an independent p-dimensional random sample vector collected at time t. The mean of the random variable $\:X$ is denoted by$\:\:\mu\:$, and its covariance matrix by$\:\:\varSigma\:$. $\:{\stackrel{-}{X}}_{t}^{D}=\frac{1}{D}{\sum\:}_{i=1}^{D}{X}_{ti}$ and $\:{S}_{t}^{D}=\frac{1}{D-1}{\sum\:}_{i=1}^{D}({X}_{ti}-{\stackrel{-}{X}}_{t})({X}_{ti}-{\stackrel{-}{X}}_{t}){\prime\:}$ represent the sample mean and sample covariance matrices at time t, respectively. It is assumed that the observed data collected at time t follows the change point model as described by Eq. (7).

7 $$\:{X}_{i}\sim\left\{\begin{array}{c}{F}_{{\mu\:}_{0}},\:i=1,\:2,\:\cdots\:,\tau\:,\\\:{F}_{\mu\:},\:\:i=\tau\:+1,\cdots\:\end{array}\right.$$

Based on the aforementioned change point model, the procedures for the D-ELEWMA chart are outlined as follows:

(1) Set the values for the smoothing coefficient $\:\lambda\:\:$and the sliding window *D*.

(2) Calculate the unilateral control limit $\:{L}_{1}$ of the D-ELEWMA control chart using a Monte Carlo simulation method repeated 10,000 times.

(3) At time t, compute $\:{Q}_{t}^{D}$ according to Eq. (4).

(4) Calculate $\:{T}_{t}^{D}$ based on Eq. (6).

(5) Compare $\:{T}_{t}^{D}$ with the control limit $\:{L}_{1}$. If $\:{T}_{t}^{D}>{L}_{1}$, issue an alarm. Otherwise, continue sampling, and shift the window to the next detection point.

## Performance evaluations

### The dataset and problem definition

**Fig. 1:**
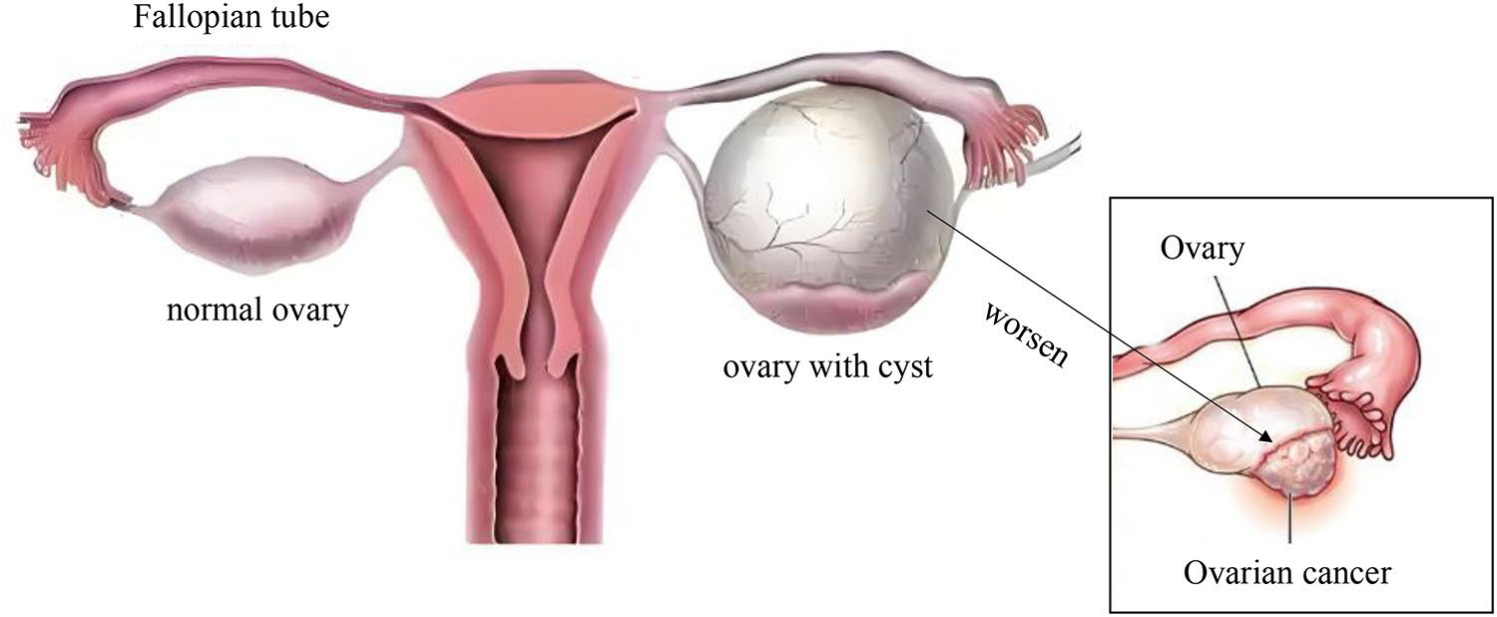
Diagram of Ovarian Tissue Lesions.

Ovarian tumors are a type of lesion originating from ovarian tissue, typically presenting as cystic or solid masses. They can be classified into three types based on their nature: benign, borderline, and malignant. The pathological changes in ovarian tissue are closely related to various factors, including genetic susceptibility, endocrine disorders, inflammatory responses, and environmental factors. Among these three tumor types, benign tumors are the most common, typically manifesting as functional ovarian cysts (such as luteal cysts and follicular cysts), serous cystadenomas, and mucinous cystadenomas. These tumors usually grow slowly and pose minimal direct health risks, with most cases being asymptomatic. However, when the cysts enlarge or compress adjacent organs, symptoms such as abdominal pain, bloating, or irregular menstruation may occur. Borderline ovarian tumors have low malignant potential and may recur or progress to malignant tumors. Malignant ovarian tumors, known as ovarian cancer, are one of the most threatening gynecological malignancies, posing a significant health risk to women. This type of tumor grows rapidly, is highly invasive, and often involves peritoneal dissemination, lymph node metastasis, and distant metastasis. Figure 1 illustrates the pathological changes in ovarian tissue. In traditional medical diagnosis, ultrasound is the preferred method for examining ovarian tumors, and combining CT or MRI can further evaluate the nature of the tumor. Additionally, tumor marker tests (such as CA125, HE4, and ROMA index) help doctors differentiate between benign and malignant tumors, although the specificity of these markers is limited.

This paper conducts a study using data from ovarian tumor resection surgeries performed at the Third Affiliated Hospital of Soochow University between July 2011 and July 2018 as a case study. The dataset is publicly available on the website: https://www.kaggle.com/datasets/saurabhshahane/predict-ovarian-cancer. A total of 349 patient samples were selected for analysis. The variables included age, 19 chemical/biological blood sample analytes, 22 general chemicals, and 6 tumor markers used for classifying benign ovarian tumors (referred to as BOT) and ovarian cancers (referred to as OC). Among these, 178 samples represented benign tumors, while 171 samples represented ovarian cancer. All patients underwent pathological diagnosis post-operation. None of the ovarian cancer patients received chemotherapy or radiation therapy before surgery.

We will monitor the 349 patients using the DELEWMA control chart. The purpose of the monitoring is to identify abnormal conditions so that an out-of-control (OOC) signal can indicate an unstable patient who requires further medical attention. In other words, the OOC signal means there is a significant difference between the current patient status and the in-control (IC) state, which is based on the empirical likelihood ratio test, and this difference may be caused by some assignable reasons that need further analysis. In practical application, the IC model is first constructed based on historical data from normal patients (i.e., Phase I analysis). Then, for each new patient (i.e., online monitoring in Phase II analysis), a model is fitted, and the estimated parameters are compared with the IC model to obtain the control chart statistics and determine whether the current state is in-control or out-of-control.

### Data processing and analysis

During the data preprocessing stage, we observed that, except for CA72-4, most indicators had a missing rate of less than 7%. However, the missing rate for CA72-4 exceeded 60%. For indicators with a low missing rate, we imputed missing values using the mean of the corresponding indicator. As for CA72-4, due to the high proportion of missing values, it had to be removed. Therefore, after preprocessing, the dataset contained 349 data points and 48 indicator variables.

**Fig. 2:**
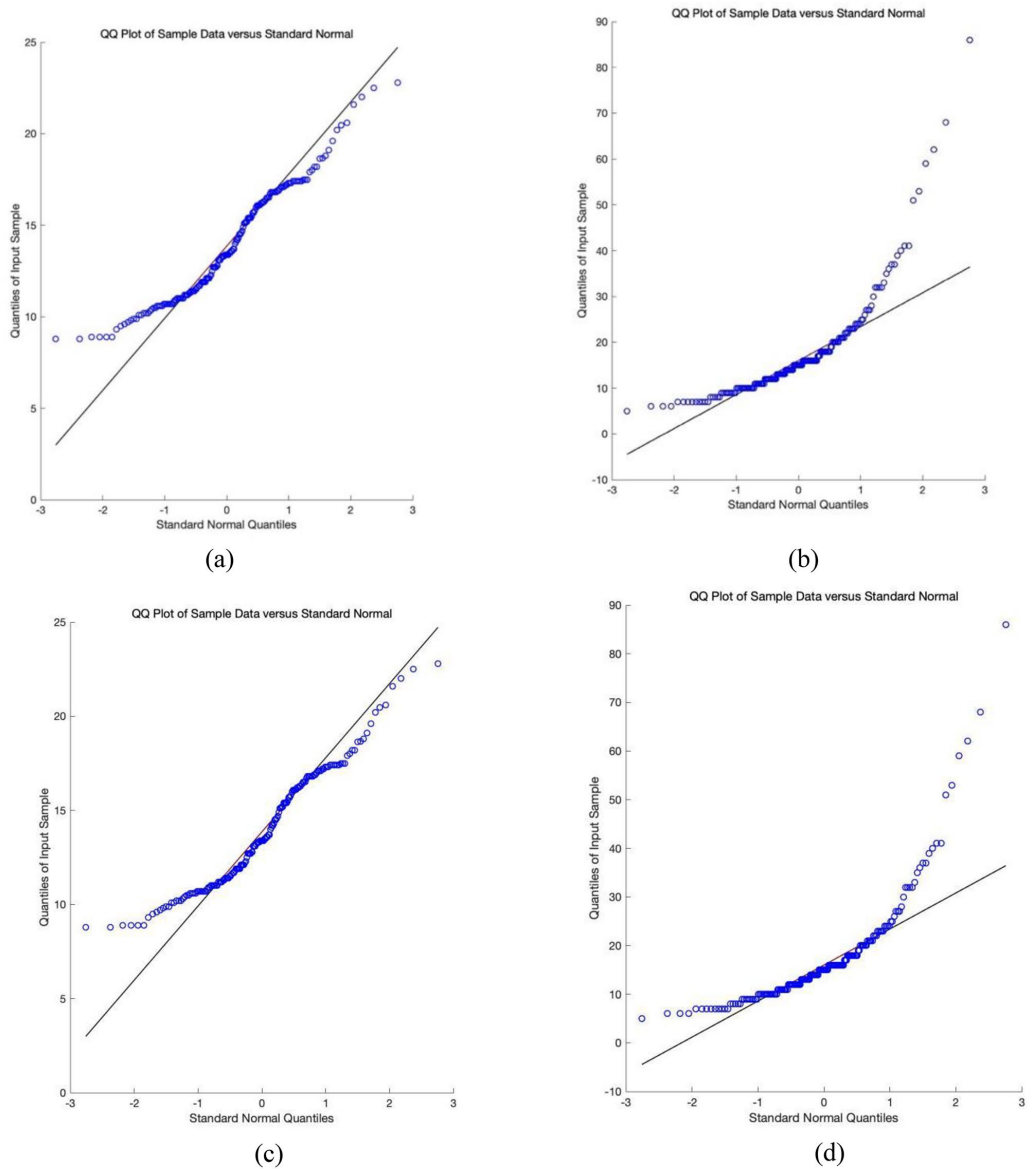
The QQ plots for Indicator Variable 6 and Indicator Variable 41. (**a**) The sixth indicator of BOT. (**b**) The 41st indicator of BOT. (**c**) The sixth indicator of OC. (**d**) The 41st indicator of OC.

**Fig. 3:**
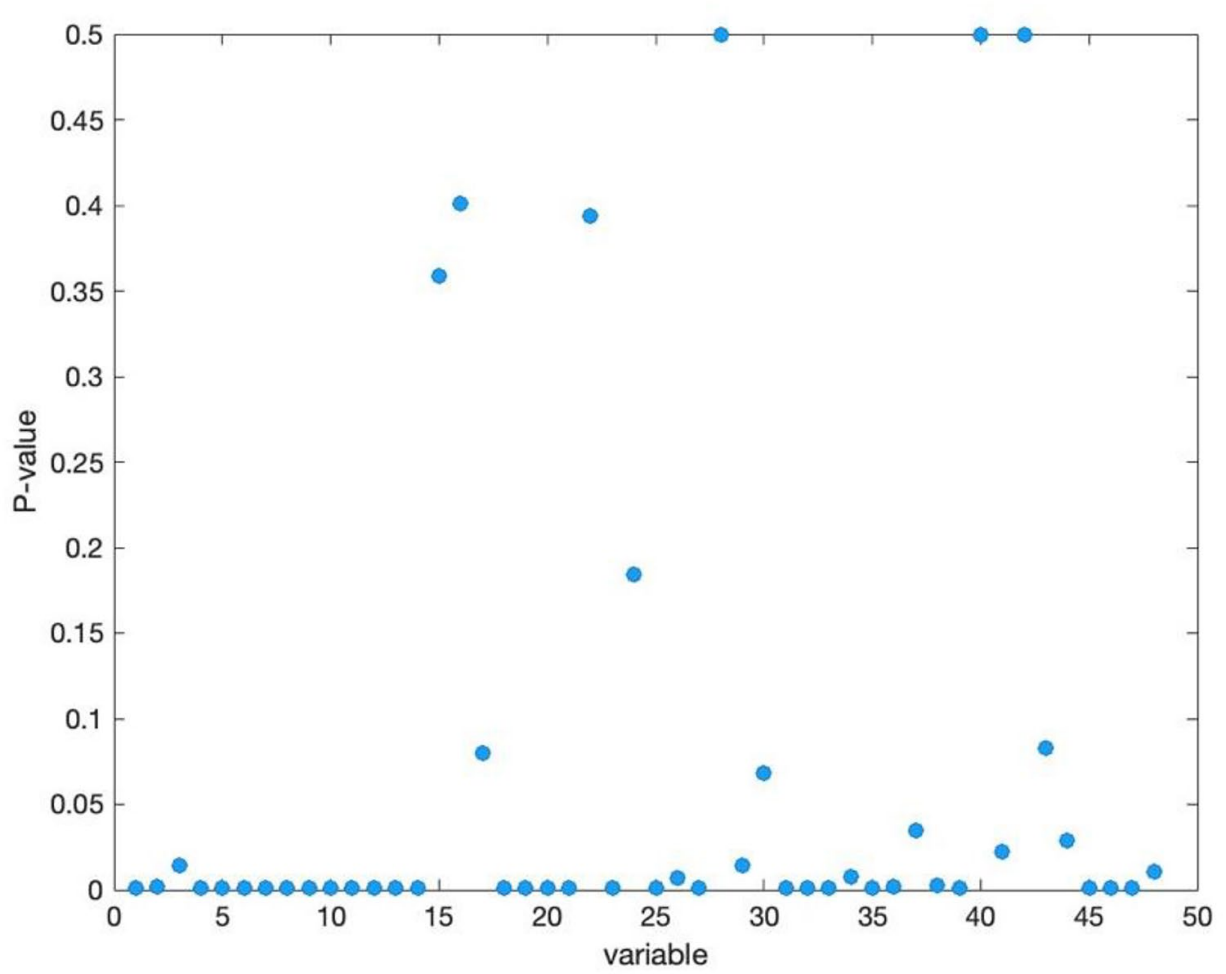
Jarque-Bera test P-values for all indicator variables.

From a data perspective, the ovarian tumor dataset is characterized by high dimensionality and a small sample size, rendering traditional univariate or multivariate control charts unsuitable. On the other hand, we performed a preliminary statistical analysis to select two indicators for benign ovarian tumors and ovarian cancer, respectively, and generated their QQ plots (see Fig. 2). The QQ plot helps assess whether data follows a specific theoretical distribution by comparing the quantiles of the data with those of the theoretical distribution. When the data points approximately align with the diagonal line, it indicates that the actual data closely matches the theoretical distribution; otherwise, it suggests a significant difference between the two. Figure 2 shows that the data points deviate from the diagonal, indicating that these two indicators do not follow a standard normal distribution, suggesting that most variables may also deviate from normality. To further investigate this hypothesis, we conducted a Jarque-Bera test for normality on 48 indicator variables. And present the p-values of the test results for each indicator in graphical form, as shown in Fig. 3. This test uses the P-value to assess the results: a P-value $\:<$ 0.05 indicates rejection of the null hypothesis, suggesting that the variable does not follow a normal distribution; otherwise, the null hypothesis is accepted. Figure 3 shows that only 10 out of 48 indicator variables have P-values $\:>$ 0.05, indicating that 80% of the variables do not conform to a normal distribution. Pseudocode 1 illustrates the procedure for calculating the Jarque-Bera test. Therefore, under these circumstances, traditional multivariate normal control charts are not applicable, making the introduction of multivariate nonparametric control charts particularly necessary.


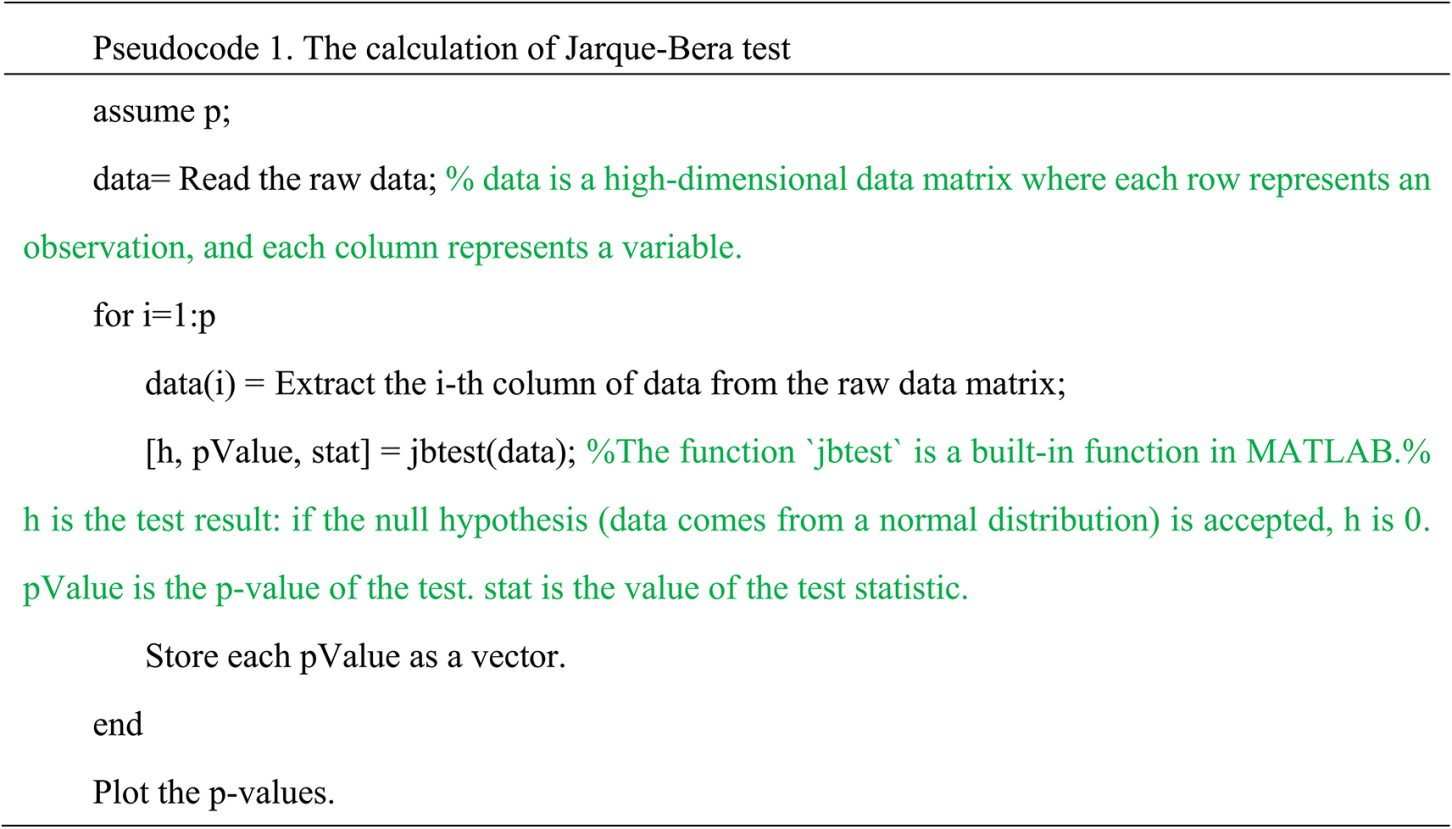


### Performance comparison through Numerical Simulation

In this section, a numerical simulation approach will be utilized to construct the D-ELEWMA model and discuss its efficiency. In evaluating the performance of control charts, the Average Run Length (ARL) is a crucial metric that reflects the average number of observations from the start of monitoring until the control chart signals an alert. $\:{ARL}_{0}$ denotes the average run length when the process is in a state of control, indicating the average number of observations before the control chart issues an alert while the process remains stable. A higher $\:{ARL}_{0}$ is desirable to reduce false alarms when the process is in control. $\:{ARL}_{1}$ represents the average run length when the process is out of control, reflecting the average number of observations from the onset of a process shift until the control chart signals an alert. A lower $\:{ARL}_{1}$ is preferred to quickly detect and respond to shifts when the process is out of control. However, these two objectives cannot be fully achieved simultaneously. Therefore, we focus on minimizing $\:{ARL}_{1}$while maintaining a stable $\:{ARL}_{0}$ to enhance the efficiency of the control chart. In this study, we use Monte Carlo simulations with a nominal $\:{ARL}_{0}$ set at 370, conducting 10,000 simulations. Furthermore, to validate the effectiveness of the model, we will compare it with the nonparametric multivariate control chart (MSEWMA) proposed by Zou et al.^39^. The data will first be dimensionally reduced before being applied to the MSEWMA control chart. To compute the control limits of the model, we generated 1000 controlled data points from the normal ranges of 46 indicators, excluding age and menopause, which include chemical/biological blood sample analytes, general chemicals, and tumor markers. The normal ranges for the corresponding 46 indicators are listed in Table 1.

**Table 1:** Relevant Information on 46 indices of blood sample analytes, General Chemicals, and tumor markers.

Abreviation	Biomarker Name	Sample type	Lower limit	Upper Limit	Unit
AFP	alpha-fetoprotein	Serum	0	7	ng/ml
AG	Anion gap	Serum	8	30	mmol/l
ALB	albumin	Serum	35	55	g/l
ALP	Alkaline phosphatase	Serum	25	130	u/l
ALT	Alanine aminotransferase	Serum	1	45	u/l
AST	Aspartate aminotransferase	Serum	6	40	u/l
BASO#	Basophil Cell Count	full blood	0	0.06	10^9/L
BASO%	Basophil Cell ratio	full blood	0	1	%
BUN	blood urea nitrogen	Serum	1.7	8.3	mmol/l
Ca	calcium	Serum	1.12	1.32	mmol/l
CA125	Carbohydrate antigen 125	Serum	0	35	U/ml
CA19-9	Carbohydrate antigen 19 − 9	Serum	0	37	U/ml
CA72-4	Carbohydrate antigen 72 − 4	Serum	0	7	U/ml
CEA	Carcinoembryonic antigen	Serum	0	5	ng/ml
CL	chlorine	Serum	99	110	mmol/l
CO2CP	carban dioxide combining Power	Serum	18	30	mmol/l
CREA	creatinine	Serum	44	144	umol/l
DBIL	direct bilirubin	Serum	1.5	7	umol/l
EO#	eosinophil count	full blood	0.02	0.52	10^9/L
EO%	eosinophil ratio	full blood	0.02	0.52	10^9/L
GGT	Gama glutamyltransferasey	Serum	3	73	u/l
GLO	globulin	Serum	20	40	g/l
GLU.	glucose	Serum	3.9	6.1	mmol/l
HCT	hematocrit	full blood	0.35	0.45	L/L
HE4	human epididymis protein 4	Serum	0	140	pmol/L
HGB	hemoglobin	full blood	110	150	g/l
IBIL	Indirect bilirubin	Serum	2	15	umol/L
K	kalium	Serum	3.5	5.3	mmol/l
LYM#	lymphocyte count	full blood	1.1	3.2	10^9/L
LYM%	lymphocyte ratio	full blood	20	50	%
MCH	Mean corpuscular hemoglubin	full blood	27	34	Pg
MCV	mean corpuscular volume	full blood	82	100	fL
Mg	magnesium	Serum	0.73	1.3	mmol/l
MONO#	mononuclear cell count	full blood	0.1	0.6	10^9/L
MONO%	monocyte ratio	full blood	3	10	%
MPV	Mean platelet volume	full blood	7.4	12.5	fL
Na	Natrium	Serum	137	147	mmol/l
NEU	neutrophil ratio	full blood	40	75	%
PCT	pthrombocytocrit	full blood	0.114	0.282	L/L
PDW	Platelet distribution width	full blood	15.5	18.1	%
PHOS	phosphorus	Serum	0.7	1.62	mmol/l
PLT	platelet count	full blood	125	350	10^9/L
RBC	Red blood cell count	full blood	3.5	5.5	10^12/L
RDW	red blood cell distribution width	full blood	10.6	15.5	%
TBIL	total bilirubin	Serum	4	19	µmol/l
TP	Total protein	Serum	60	82	g/l
UA	urie acid	Serum	90	450	µmol/l

#### The MSEWMA control chart

The process overview of the MSEWMA control chart proposed by Zou et al.^39^ is as follows:

(1) The estimation of $\:({\theta\:}_{0},\:{A}_{0})$ using controlled historical samples is performed as follows: $\:{\theta\:}_{0}={\mu\:}_{0}$ and $\:{A}_{0}{\prime\:}{A}_{0}={p}^{-1}trace\left({{\Sigma\:}}_{0}\right){{\Sigma\:}}_{0}^{-1}$, where $\:{\mu\:}_{0}$ and $\:{{\Sigma\:}}_{0}$ represent the sample mean and sample covariance matrix corresponding to the controlled historical samples, respectively.

(2) The observations $\:{x}_{i}={\left({x}_{i1},{x}_{i2},\cdots\:,{x}_{iD}\right)}^{{\prime\:}},\:i=\text{1,2},\cdots\:$, collected online are subjected to the following standardization process:

8 $$\:{v}_{i}=\frac{1}{D}\sum\:_{i=1}^{D}\frac{{A}_{0}({x}_{ij}-{\theta\:}_{0})}{\left|\right|{A}_{0}({x}_{ij}-{\theta\:}_{0})\left|\right|}.$$

(3) Incorporating $\:{v}_{i}$ into the EWMA model:

9 $$\:{w}_{i}=\left(1-\lambda\:\right){w}_{i-1}+\lambda\:{v}_{i},\:{v}_{0}=0.$$

(4) The monitoring statistic they constructed is:

10 $$\:{S}_{i}=\frac{2-\lambda\:}{\lambda\:}p{w}_{i}^{{\prime\:}{w}_{i}}>{L}_{2},$$

where, $\:{L}_{2}$ represents the control limit of the MSEWMA.

#### Parameter setting

First, the choice of the smoothing coefficient λ is considered. Typically, a relatively small λ value enhances the detection capability for minor shifts, while a larger λ value is more effective in identifying significant shifts. Therefore, in accordance with the majority of the literature, we have selected λ values of 0.05, 0.1, and 0.2 for our simulations.

And then, the selection of specific parameters within the monitoring statistic is considered. Based on Theorem 1 and sensitivity analysis conducted in Cui^32^, it is evident that for D-ELEWMA control, optimal choices for certain parameters in the expression of the factor $\:{a}_{n}$ of the added artificial virtual point are as follows: $\:{l}_{n}={n}^{5/4}logn$, $\:{k}_{n}={(p/logp)}^{1/2}$ and $\:\gamma\:={\left(1,\:1,\cdots\:,\:1\right)}^{{\prime\:}}/\sqrt{p}$.

#### Calculation and Simulation results

To apply the MSEWMA control chart, we selected the most representative 6 features using the Minimum Redundancy Maximum Relevance (MRMR) feature selection method proposed by Lu et al.^40^: AFP, CEA, HE4, CA19-9, LYM%, and CO2CP.

The upper control limits are denoted as UCL_DEL_​ and UCL_MS_​. These control limits can be obtained by performing 10,000 Monte Carlo simulations under a predefined zero-hypothesis average run length $\:{(ARL}_{0}=370$) or a Type I error rate of 0.0027. Pseudocode 2 illustrates the procedure for calculating UCL_DEL_​, and similar procedures can be followed for the UCL_MS_​. The calculated values of UCLDEL​ and UCLMS for sliding window sizes D of 6, 8, and 10, along with the corresponding $\:{ARL}_{0}$​ and SDRL, are presented in Table 2.
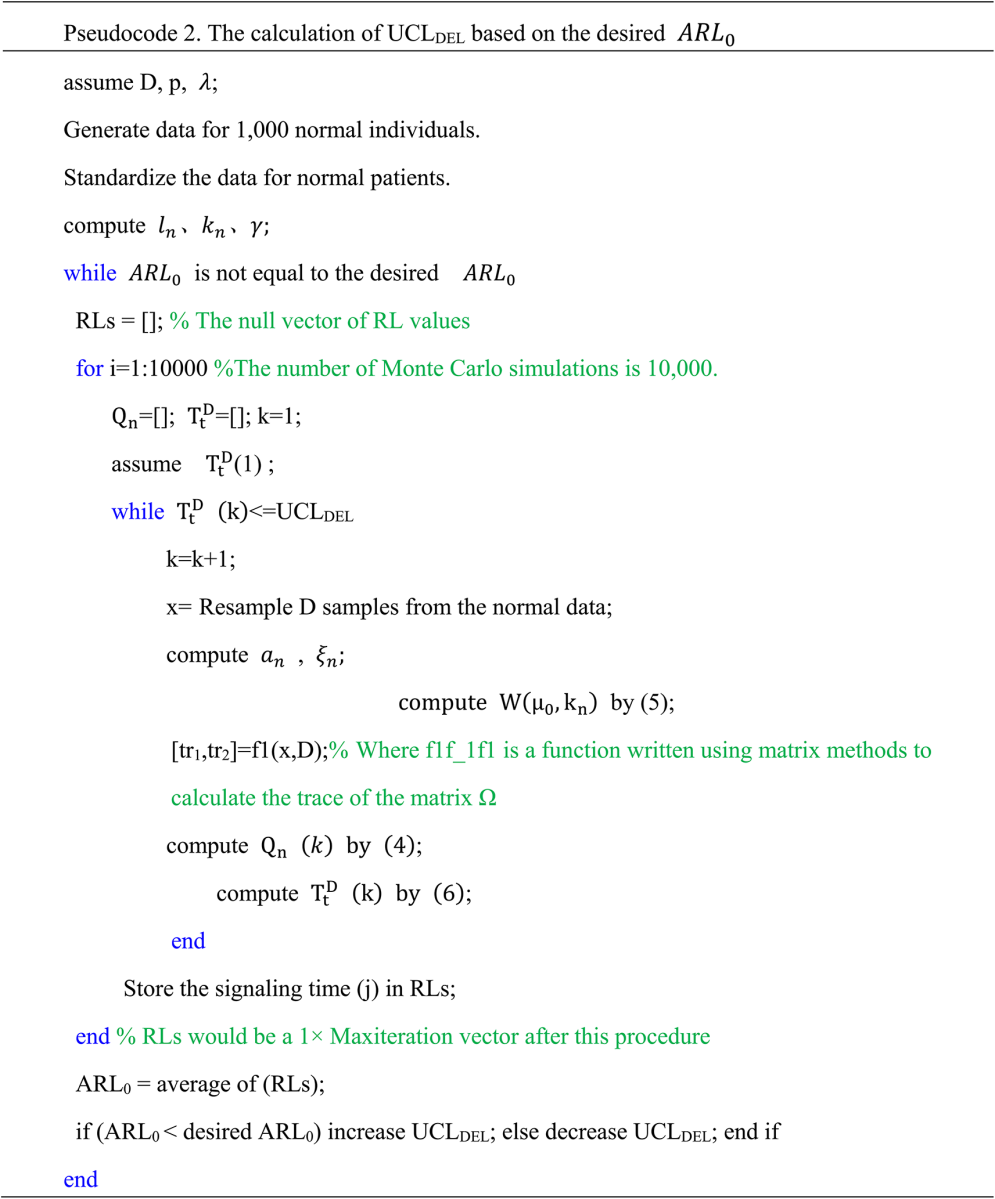


**Table 2:** The control limits, $\:{ARL}_{0}$, and SDRL for D-ELEWMA and MSEWMA.

	D-ELEWMA				MSEWMA			
		$\:\lambda\:$				$\:\lambda\:$		
		0.05	0.1	0.2		0.05	0.1	0.2
D = 10	$\:{L}_{1}$	−0.2018	−0.032	0.248	$\:{L}_{2}$	1.65	1.789	1.88
	$\:{ARL}_{0}$	371.1772	370.2585	370.0894	$\:{ARL}_{0}$	370.3894	371.1409	370.3352
	SDRL	362.6909	363.5753	359.4885	SDRL	351.2104	365.4463	356.1839
D = 8	$\:{L}_{1}$	−0.435	−0.2888	−0.042	$\:{L}_{2}$	2.065	2.235	2.336
	$\:{ARL}_{0}$	369.1469	371.905	370.4096	$\:{ARL}_{0}$	370.5934	369.3943	372.9111
	SDRL	361.9662	369.053	371.0973	SDRL	356.0978	360.6731	367.4085
D = 6	$\:{L}_{1}$	−0.8447	−0.7319	−0.5507	$\:{L}_{2}$	2.743	2.9714	3.1038
	$\:{ARL}_{0}$	370.8533	370.9939	370.1637	$\:{ARL}_{0}$	370.8303	371.2587	368.9641
	SDRL	359.7683	366.8746	364.6483	SDRL	349.2686	359.5137	360.8462

**Fig. 4:**
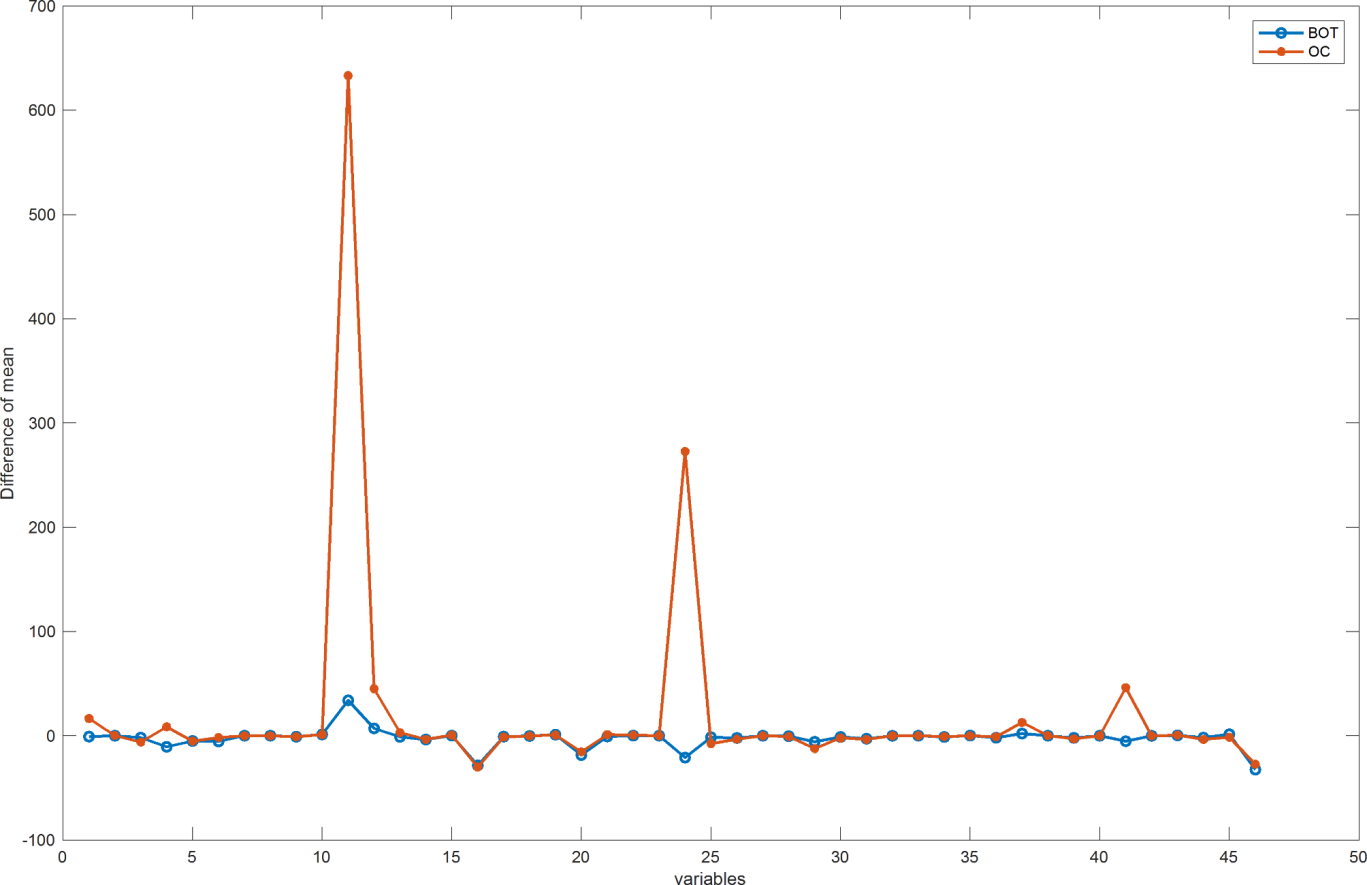
The mean differences between controlled data and BOT data, OC data.

To simulate a more realistic $\:{ARL}_{1}$, we plotted the mean differences between 1000 controlled data and 178 BOT data and 171 OC data, as shown in Fig. 4.

From Fig. 4, it can be observed that the mean shift approximately occurs at variables 1, 4, 11, 12, 16, 20, 29, 36, 42, and 46. We simultaneously applied shifts following normal distributions with mean magnitudes of 0.3, 0.5, and 0.8, and variances of 1 to these variables. At this point, only three variables, AFP, CA19-9, and CO2CP, exhibited shifts in the MSEWMA control chart. Pseudocode 3 shows how an artificial shift is applied to the mean of the normal data.
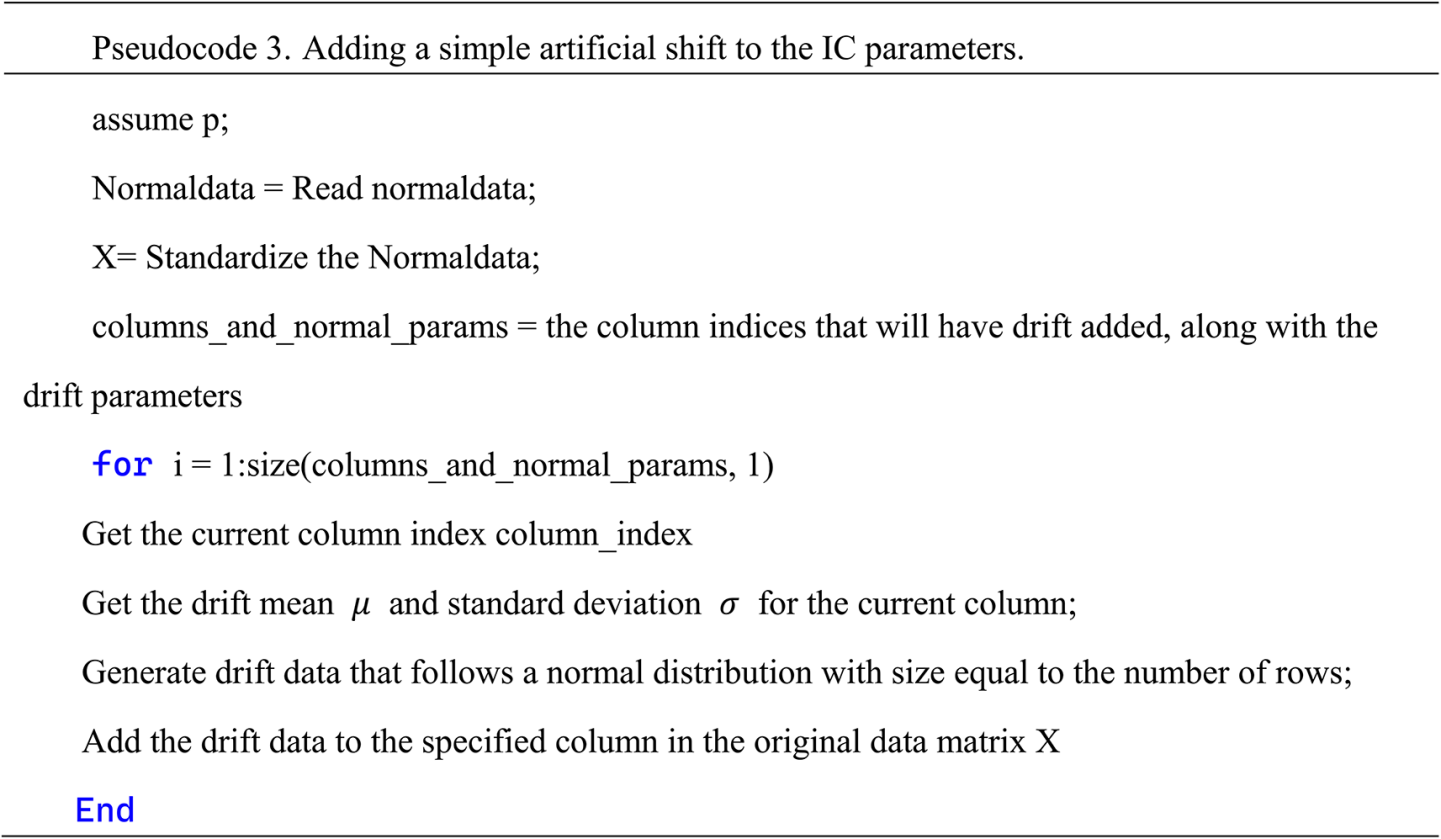


Subsequently, we calculated the $\:{ARL}_{1}$ for both the D-ELEWMA and the MSEWMA control chart, as shown in Table 3. The calculation of $\:{ARL}_{1}$ is similar to Pseudocode 1, with only slight modifications. For example, remove the outermost loop and change the data reading step to read the anomalous data generated in Pseudocode 3 instead of the normal data.

**Table 3:** The $\:{ARL}_{1}$ values for D-ELEWMA and MSEWMA control chart with $\:{ARL}_{0}=370$, $\:D=\{\text{6,8},10\}$, $\:\lambda\:=\left\{\text{0.05,0.1,0.2}\right\}$, shift $\:=\left\{\text{0.3,0.5,0.8}\right\}$.

			$$\:\lambda\:$$								
			0.05			0.1			0.2		
			0.3	0.5	0.8	0.3	0.5	0.8	0.3	0.5	0.8
D-ELEWMA	D = 10	$\:{ARL}_{1}$	12.629	4.1797	1.978	12.1941	3.7176	1.7391	13.0415	3.3858	1.534
		SDRL	7.1795	1.6946	0.6757	8.0356	1.6954	0.6483	10.0045	1.7583	0.6186
	D = 8	$\:{ARL}_{1}$	18.3883	5.5647	2.5079	18.2298	4.9929	2.1902	20.6295	4.6871	1.926
		SDRL	11.4811	2.3827	0.8641	13.1359	2.4403	0.8241	16.9049	2.6586	0.815
	D = 6	$\:{ARL}_{1}$	33.195	8.368	3.4269	36.5627	7.933	3.0414	42.9937	7.754	2.6995
		SDRL	24.3782	4.0688	1.2411	31.0497	4.517	1.1972	39.5068	5.1158	1.2165
MSEWMA	D = 10	$\:{ARL}_{1}$	13.7417	7.6735	5.0902	12.1017	6.1785	4.0179	12.3032	5.2556	3.2415
		SDRL	4.3325	1.5943	0.7972	4.974	1.6006	0.7674	7.1423	1.8281	0.7549
	D = 8	$\:{ARL}_{1}$	15.8594	8.6417	5.6968	14.1574	7.0735	4.5069	15.2473	6.1277	3.6595
		SDRL	5.3655	1.9379	0.9383	6.3728	1.989	0.886	9.8247	2.3577	0.9258
	D = 6	$\:{ARL}_{1}$	18.7951	10.0923	6.5688	17.7317	8.4252	5.2521	20.4211	7.6503	4.35
		SDRL	7.0713	2.5043	1.1875	8.9394	2.659	1.1506	14.4275	0.3328	1.2362

As shown in Table 3, when the sliding window size $\:D=10,$ regardless of the value of $\:\lambda\:$ and the magnitude of the shifts, the $\:{ARL}_{1}$ of the D-ELEWMA control chart is significantly lower than that of the MSEWMA control chart. This indicates that the D-ELEWMA control chart would detect changes in ovarian cyst data faster and issue out-of-control alarms earlier than the MSEWMA control chart under these conditions. When $\:D=8$ or $\:D=6$, the D-ELEWMA control chart outperforms the MSEWMA control chart in monitoring and detecting significant shifts, indicating that the D-ELEWMA control chart is more effective for ovarian cyst data with larger variations in relevant indicators.

## Application of D-ELEWMA and MSEWMA control chart to ovarian tumor data

**Fig. 5:**
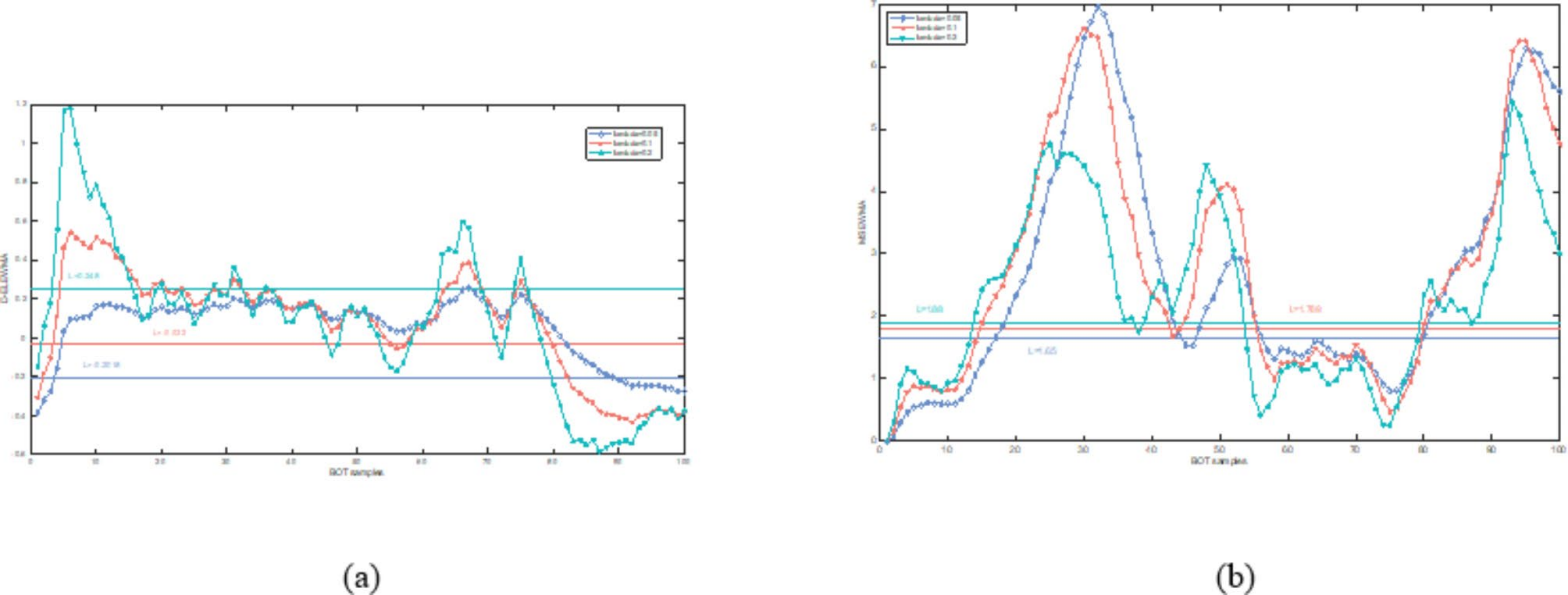
When D = 10, the D-ELEWMA control chart and the MSEWMA control chart plotted for BOT data (only the first 100 data points are shown. (**a**) The D-ELEWMA chart. (**b**) The MSEWMA chart.

**Fig. 6:**
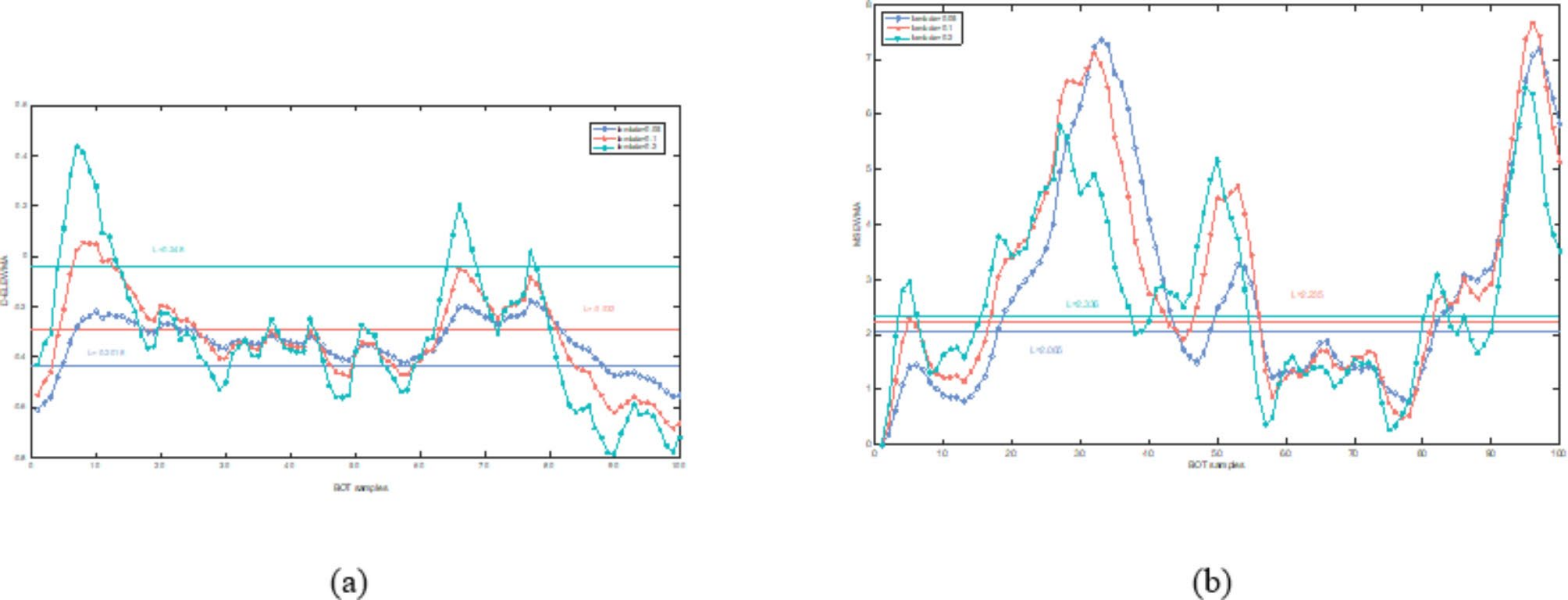
When D = 8, the D-ELEWMA control chart and the MSEWMA control chart plotted for BOT data (only the first 100 data points are shown. (**a**) The D-ELEWMA chart. (**b**) The MSEWMA chart.

**Fig. 7:**
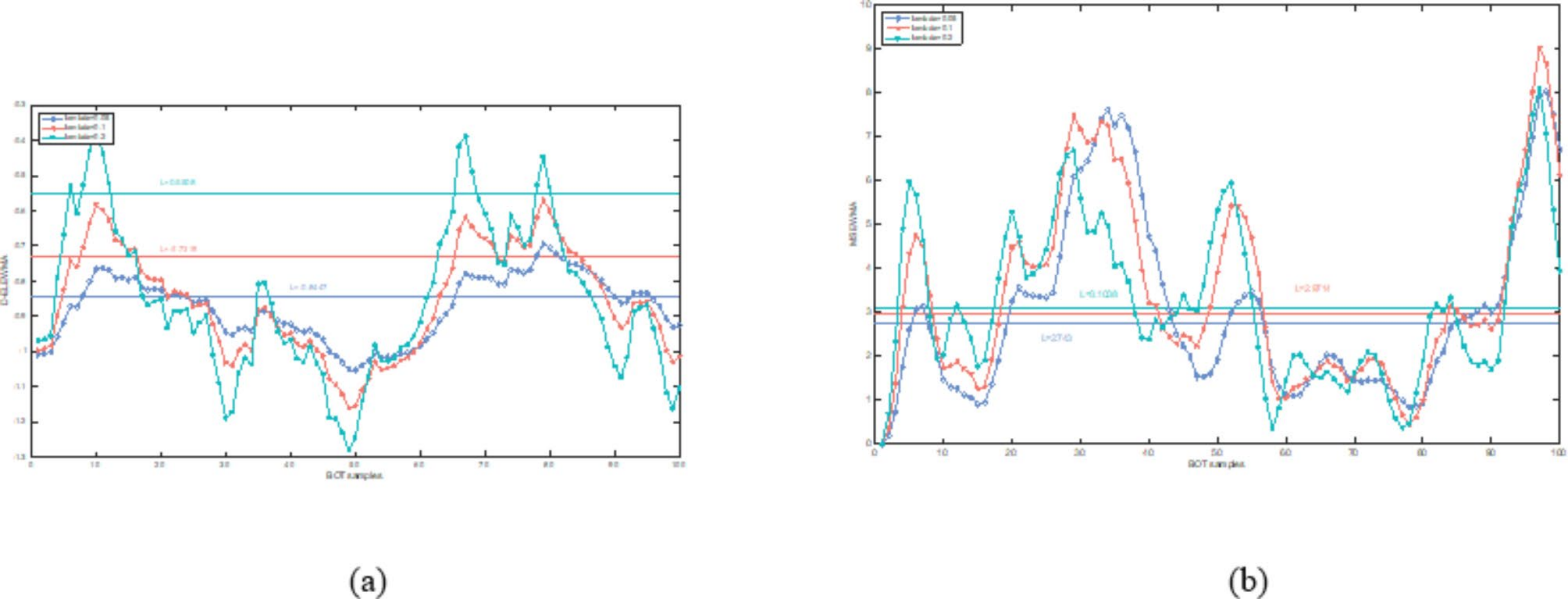
When D = 6, the D-ELEWMA control chart and the MSEWMA control chart plotted for BOT data (only the first 100 data points are shown. (**a**) The D-ELEWMA chart. (**b**) The MSEWMA chart.

**Fig. 8:**
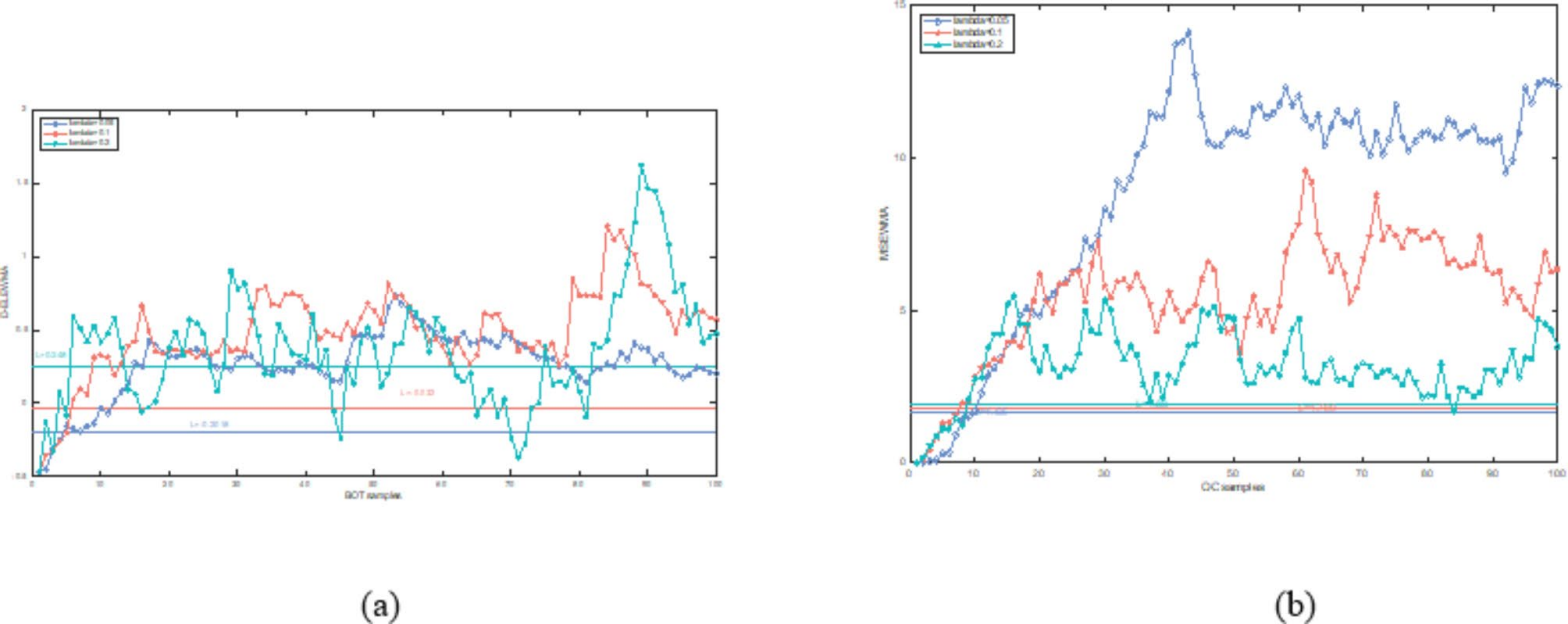
When D = 10, the D-ELEWMA control chart and the MSEWMA control chart plotted for OC data (only the first 100 data points are shown. (**a**) The D-ELEWMA chart. (**b**) The MSEWMA chart.

**Fig. 9:**
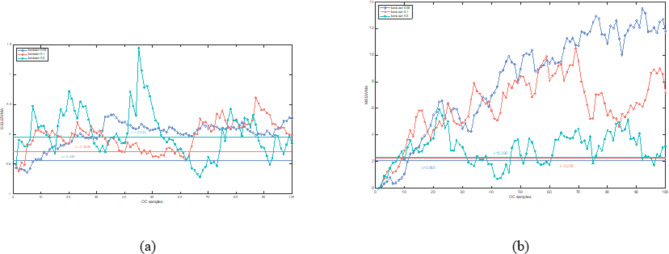
When D = 8, the D-ELEWMA control chart and the MSEWMA control chart plotted for OC data (only the first 100 data points are shown. (**a**) The D-ELEWMA chart. (**b**) The MSEWMA chart.

**Fig. 10:**
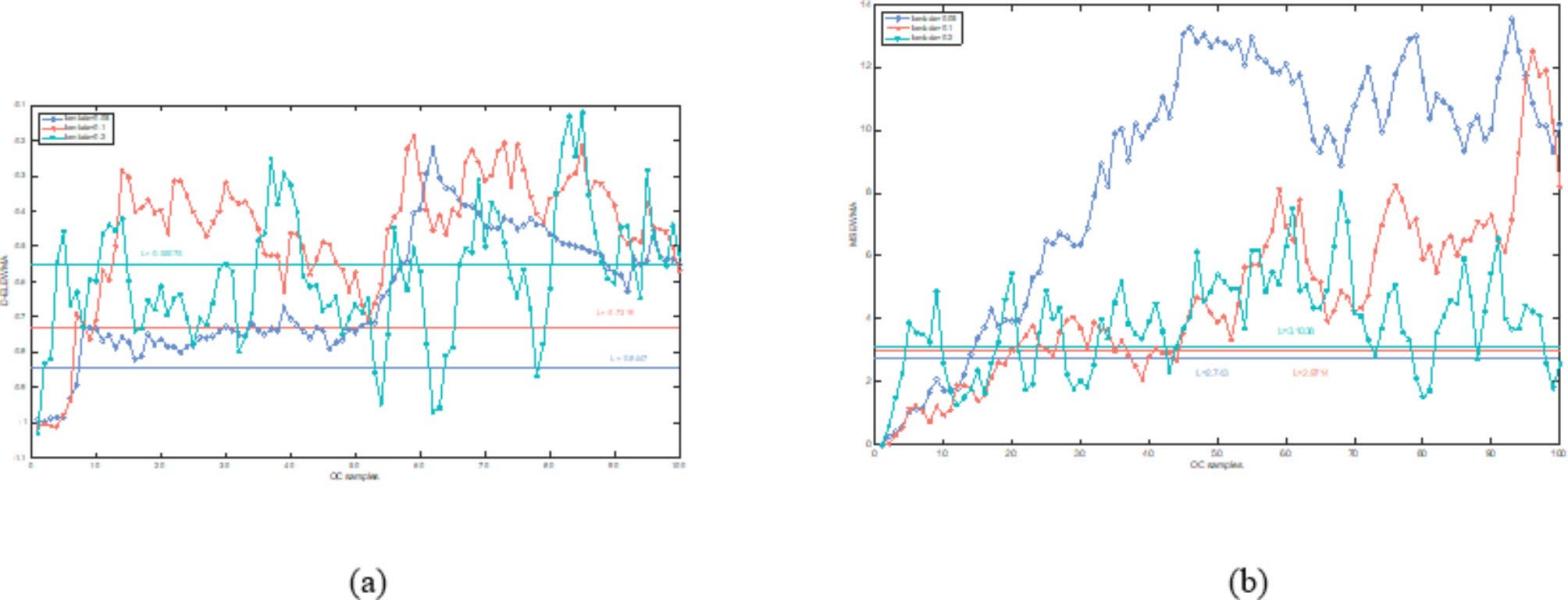
When D = 6, the D-ELEWMA control chart and the MSEWMA control chart plotted for OC data (only the first 100 data points are shown. (**a**) The D-ELEWMA chart. (**b**) The MSEWMA chart.

In this section, both the D-ELEWMA and MSEWMA control charts were applied to both BOT data and OC data, with a nominal $\:{ARL}_{0}$ of 370. Therefore, based on the control lines shown in Table 2, we plotted control charts with varying smoothing coefficient $\:\lambda\:$ values and sliding window sizes, as shown in Figs. 5, 6, 7, 8, 9 and 10. In the control chart, there are three lines: the center line, the upper control limit, and the lower control limit. The points plotted in the chart represent the values of the DELEWMA and MSEWMA statistics. The upper and lower control limits are shown in Table 2.

As shown in Fig. 5, with the sliding window size set to 10, when $\:\lambda\:$ is 0.05, the D-ELEWMA control chart issued an alarm for process out-of-control upon monitoring the fourth Benign Ovarian Tumor (BOT) sample point; in comparison, the MSEWMA control chart did not issue an alarm until it had monitored the fourteenth sample point. When $\:\lambda\:$ was increased to 0.1, the D-ELEWMA control chart alerted at the monitoring of the fifth sample point, whereas the MSEWMA control chart issued its alarm at the fifteenth sample point. Upon elevating $\:\lambda\:$ to 0.2, the D-ELEWMA control chart once again alerted at the fourth sample point, while the MSEWMA control chart issued an alarm at the seventeenth sample point. These results suggest that the D-ELEWMA control chart is more sensitive to detecting anomalies in BOT data compared to the MSEWMA control chart and provides an earlier warning signal.

As shown in Figs. 6 and 7, when $\:D=8$, the D-ELEWMA control chart outperforms the MSEWMA control chart in monitoring BOT data only when $\:\lambda\:\:=0.2$. At this point, the D-ELEWMA control chart issues an out-of-control alarm upon monitoring the fifth sample point, whereas the MSEWMA control chart does so upon monitoring the eighteenth sample point. However, when $\:D=6$, regardless of the value of $\:\lambda\:$, the MSEWMA control chart slightly outperforms the D-ELEWMA control chart in monitoring BOT data.

As shown in Figs. 8, 9 and 10, regardless of the values of and $\:\lambda\:$, the D-ELEWMA control chart consistently outperforms the MSEWMA control chart in monitoring OC data. The D-ELEWMA chart detects anomalies in the data more rapidly and provides earlier alerts for changes in relevant indicators. If physicians can detect these anomalies and initiate interventions upon receiving an alert, they can more effectively track disease progression, improve the efficiency of resource utilization, support clinical decision-making, and enhance patient self-management and health education. This approach supports early prevention and intervention for the disease.

In summary, when monitoring BOT data, the D-ELEWMA control chart is markedly superior to the MSEWMA control chart only when $\:D=10$. Conversely, in the monitoring of OC data, the D-ELEWMA consistently outperforms the MSEWMA under any settings. The corresponding indicator data for patients with benign ovarian cysts exhibit relatively minor variations compared to the indicator data for healthy females, which corresponds to the small shifts in the simulation scenarios. In contrast, the indicator data for ovarian cancer patients experience relatively significant changes, corresponding to the medium and large shifts in the simulation scenarios. This indicates that the results obtained from applying the D-ELEWMA and MSEWMA control charts to actual data are consistent with the outcomes of numerical simulations.

## Conclusions

As one of the most common cancers among women, the use of statistical methods for online monitoring and early warning of benign and malignant ovarian tumors has had a profound impact on the lives of many. Therefore, enhancing the accuracy of early diagnosis and detection of ovarian cancer is of significant importance to us. Utilizing statistical process control (SPC) tools, we systematically investigated methods for monitoring and early warning of ovarian cancer, which included patient demographics, routine blood tests, general chemistry, and the selection of the most relevant tumor marker features.

The empirical likelihood ratio test we employed not only endowed the constructed control chart with good statistical performance but also enabled monitoring of high-dimensional ovarian tumor data with an unknown sample distribution. This approach addressed some of the shortcomings associated with certain parametric or multivariate control charts. Additionally, we integrated the smoothing parameter lambda of the EWMA to assign different weights to historical and current data, thus preventing the loss of historical information. Furthermore, we employed a sliding window model to monitor ovarian cyst data as a single data stream, which circumvents the poor monitoring effects caused by the limited amount of data in subgroup data flow control charts. Monte Carlo numerical simulation results demonstrated that, compared to dimensionality reduction followed by the application of multivariate control charts, our high-dimensional nonparametric control chart was significantly faster in detecting data changes when $\:\varvec{D}=10$. In other settings of , our method outperformed in monitoring medium to large shifts. Finally, taking the tumor resection data from the Third Affiliated Hospital of Soochow University as an example, we conducted online monitoring and early warning analysis for benign ovarian cyst and ovarian cancer data, with the monitoring and early warning results being consistent with the numerical simulation outcomes. When a control chart signals an anomaly, it indicates that the indicators for benign ovarian cysts and ovarian cancer patients have deviated from the normal range compared to healthy individuals. The appearance of a control chart signal is useful for the early detection of potential disease changes; however, it cannot directly confirm the presence of a tumor or determine its exact location. Further evaluation of the patient’s health status, combined with other clinical information and diagnostic tools, is necessary for a comprehensive analysis and adjustment of the treatment plan.

Future work for this study could involve developing a tool that integrates the high-dimensional nonparametric monitoring model developed in this research and applies it to a large patient population for further validation.

## Electronic supplementary material

Below is the link to the electronic supplementary material.

Supplementary Material 1

## Data Availability

The data used to support the findings of this study are available from the corresponding author upon request.
